# One-shot platelet-rich plasma (PRP) injection is non-inferior to extracorporeal shockwave therapy in the management of supraspinatus tendinosis

**DOI:** 10.1007/s12306-023-00778-x

**Published:** 2023-03-11

**Authors:** L. Moretti, D. Bizzoca, G. D. Cassano, M. Coviello, A. Franchini, B. Moretti

**Affiliations:** 1 Department DiBraiN, School of Medicine, University of Bari “Aldo Moro”, AOU Consorziale Policlinico, Piazza Giulio Cesare 11, 70124 Bari, Italy; 2 PhD Course in Public Health, Clinical Medicine, and Oncology, University of Bari “Aldo Moro”, Piazza Giulio Cesare 11, 70100 Bari, Italy

**Keywords:** Shoulder, Supraspinatus tendinosis, PRP, PRP injection, Amateur athletes, ESWT, Rotator cuff tendinopathy

## Abstract

**Purpose:**

Supraspinatus tendinosis (ST) refers to the intratendinous degeneration of the supraspinatus tendon. Platelet-Rich Plasma (PRP) is one of the possible conservative treatments for supraspinatus tendinosis. This prospective observational study aims to evaluate the efficacy and safety of a single ultrasound-guided PRP injection in the treatment of supraspinatus tendinosis and to assess its non-inferiority to the widely used shockwave therapy.

**Methods:**

Seventy-two amateur athletes (35 male, mean age: 43.75 ± 10.82, range 21–58 years old) with ST were finally included in the study. All the patients underwent clinical evaluation at baseline, (T0) and at 1-month (T1), 3-month (T2) and 6-month (T3) follow-up using the following clinical scales: the Visual Analogue Scale for pain (VAS), Constant Score and the Disabilities of the Arm, Shoulder and Hand Score (DASH). A T0 and T3 ultrasound examination was also performed. The findings observed in the recruited patients were compared to the clinical results observed in a retrospective control group made up of 70 patients (32 male, mean age = 41.29 ± 13.85, range 20–65 years old) treated by extracorporeal shockwave therapy (ESWT).

**Results:**

VAS, DASH and Constant scores significantly improved from T0 to T1; the improvement in clinical scores was kept until T3. No local nor systemic adverse events were observed. An improvement in the tendon structure was observed on ultrasound examination. PRP showed a non-statistical inferiority, in terms of efficacy and safety, compared to ESWT.

**Conclusion:**

The PRP one-shot injection is a valid conservative treatment to reduce pain, and improve both quality of life and functional scores in patients with supraspinatus tendinosis. Furthermore, the PRP intratendinous one-shot injection showed a non-inferiority in terms of efficacy at the 6-month follow-up, compared to ESWT.

## Introduction

Shoulder disorders are a common musculoskeletal disease, with a 1-year prevalence of 47% and a lifetime prevalence of up to 70% [1]. Rotator cuff tendinopathy, causing pain and weakness during external rotation and elevation, is one of the most common causes of shoulder pain [2].

The supraspinatus tendon is the one that most frequently undergoes damage. The spectrum of lesions can include tendon inflammation, tendon degeneration and rupture, which can be partial or complete. Supraspinatus Tendinosis (ST) refers to the intratendinous degeneration of the supraspinatus tendon. A combination of extrinsic mechanical compression (i.e., narrowing of the subacromial space) and tendon overuse/overload (i.e., repetitive overhead are shown to be the major mechanism of tendinopathy [3]. The management of rotator cuff tendinopathy is mainly conservative unless the patient has a complete tendon tear or high functional demands [3].

Several options are available to conservatively treat supraspinatus tendinosis, including manual therapy [4], therapeutic ultrasound [5], extracorporeal shockwave therapy (ESWT) [6], low-level laser therapy (LLT), transcutaneous electrical nerve stimulation (TENS), pulsed electromagnetic field therapy (PEMFs) [7, 27, 28, 33] and injection therapy with corticosteroids, hyaluronic acid and Platelet-Rich Plasma (PRP).

ESWT has been successfully used to achieve pain relief in several musculoskeletal disorders, including rotator cuff pathology [29, 30]. In a recent randomized clinical trial, recruiting 84 patients suffering from rotator cuff tendonitis, Li et al. reported ESWT showed significantly greater efficacy in shoulder pain relief, compared to the placebo group, at 4-week and 8-week follow-up [30].

Corticosteroid injections, widely used in the past also in the management of rotator cuff tendinopathy, should be currently used exclusively for adhesive capsulitis and avoided in case of tendon pathology since they predispose to further tendon tears [8]. Moreover, corticosteroids bring satisfactory results only in the short term as evidenced in the meta-analysis by Lin et al. [9].

PRP is a valid alternative to corticosteroid injections. It is an autologous blood product containing a high percentage of various growth factors (GFs), such as vascular endothelial growth factor, transforming growth factor- b, epidermal growth factor, fibroblast growth factor, and platelet-derived growth factor [10, 31]. GFs and cytokines, released by the platelets after being damaged by an injury or pathology, might be involved in modulating the inflammatory processes contributing to the tissue structures’ preservation or regeneration [10, 31]. Furthermore, the infiltration of PRP has positive effects on pain and functional limitations [11].

This study aims to assess the clinical effects of PRP one-shot injection in a patient affected by Supraspinatus Tendinosis (ST) at 6 months follow-up and its non-inferiority to the widely used shockwave therapy.

## Materials and methods

We designed a prospective observational study, approved by the local Ethics Committee. The subjects enrolled signed informed consent. The study was also registered on ClinicalTrials.gov (NCT04851951).

**Fig. 1:**
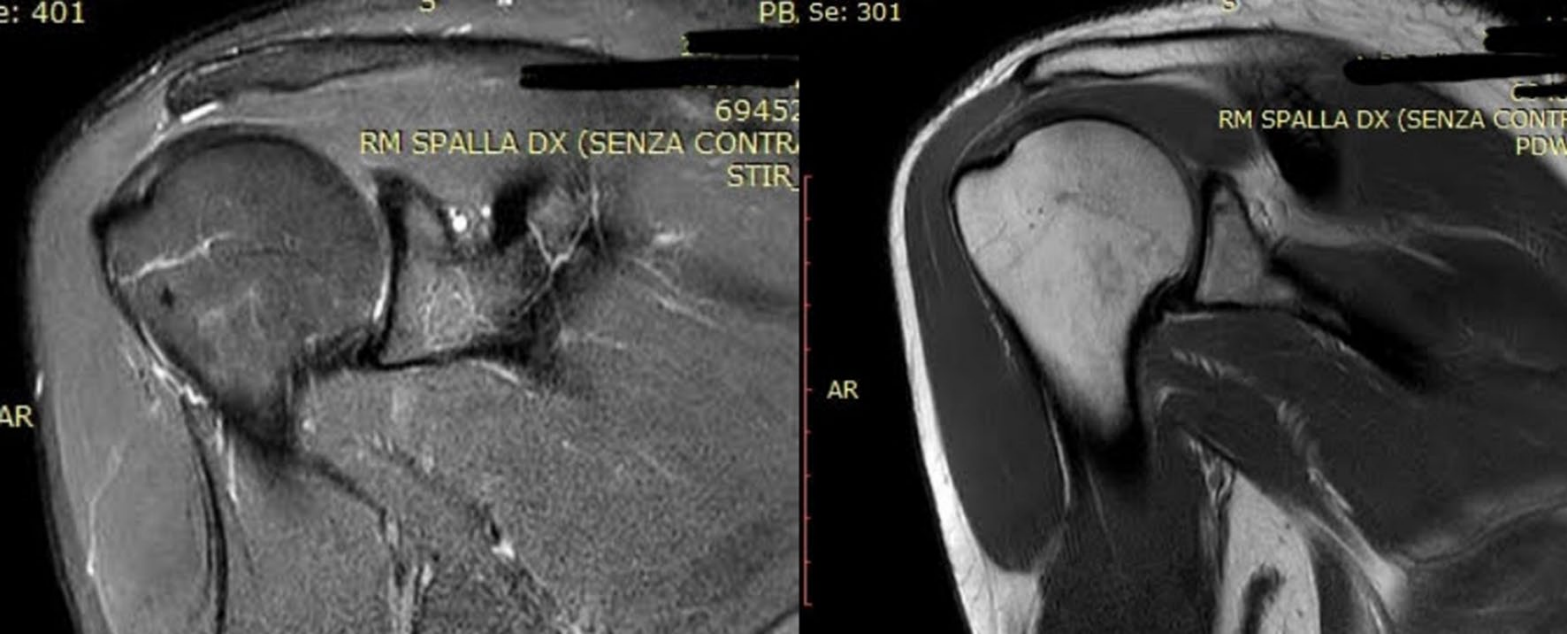
MRI Coronal STIR-weighted image and PD-weighted image of the supraspinatus tendons of an enrolled patient at T0

Seventy-five (75) patients referring to the Orthopaedic and Trauma Unit of the local University Hospital between January 2018 and July 2020 with Supraspinatus tendinosis were prospectively recruited. Figure 1 shows an MRI Coronal STIR-weighted image and PD-weighted image of the supraspinatus tendon of an enrolled patient at T0.

Inclusion criteria: age > 18 years old and < 65 years old; supraspinatus tendinosis detected on Magnetic Resonance Imaging (MRI); amateur athletes playing sports involving the upper limb (i.e., tennis, paddle tennis, squash, handball, basketball, volleyball, and swim). Exclusion criteria: supraspinatus rupture; previous shoulder surgery; rotator cuff tears secondary to fracture; Hemoglobin < 7 g/dL or Platelets < 30,000 μL; active infection; pregnancy; patients with hematologic or oncologic diseases.

All patients underwent at recruitment routine blood tests, including a complete blood count and screening for transmission-transmitted diseases (i.e., HIV, HBV, and HCV).

All tests have been performed by the same researchers (two orthopedic surgeons with more than ten years of experience in shoulder surgery), who tested all patients.

The evaluation times were T0 (recruitment), T1 (one month after the injection), T2 (three months after the last injection) and T3 (six months after the last injection). At recruitment (T0), epidemiological data (sex, age, weight, and height) and clinical history (level of activity, type of injury, previous therapies, instrumental examinations, and co-morbidities) were collected. The Constant Score [12], the Disabilities of the Arm, Shoulder and Hand Score (DASH) [13] and the Visual Analogue Scale (VAS) were administered at each clinical assessment. Shoulder physical examination with the evaluation of active and passive Range of Motion (ROM) and specific tests was also performed at each follow-up visit. An ultrasound examination was also performed before the PRP injection and at T3.

The PRP concentrate used for intratendinous injection was prepared, with good manufacturing practices at the Immunohematology Unit of Bari University Hospital, from autologous venous blood apheresis. All the patients were advised to fast for 10 h before the blood collection, to avoid any effects of food intake on PRP concentrate, meanwhile water intake was not restricted. Venous blood was drawn from the antecubital vein of each patient, then it was centrifuged with Arthrex Angel System (Arthrex, Naples, Florida, USA) to separate the blood, the plasma, the buffy coat and residual red blood cells (RBCs).

A single injection of PRP (7% concentrate) ml 4 was made in the Supraspinatus tendon and the subacromial space under ultrasound guidance. All the procedures were carried out in an aseptic condition. The patient was observed for 30 min under medical care after the procedures and then they were discharged if no complications appear. A post-treatment therapy was prescribed to each patient consisting of an antibiotic cycle, functional rest for 24–48 h, paracetamol (in case of post-procedural pain) and local cryotherapy. No adverse event was observed in the treated data.

The retrospective control group received ESWT that was applied using a shockwave device (Minilith, Storz®) equipped with in-line ultrasound-guidance Aloka SSD 900 (Aloka Co., Ltd. Tokyo, Japan), with 3000 pulses, giving 1000 impulses for session one per week, of 0.11 mJ/mm^2^ at a frequency of 15 Hz and pressure was set at 3 bar. The VAS mean score was compared in both groups at all follow-ups.

All patients receiving PRP injections underwent diagnostic US of the painful shoulder with a US scanner (Sonosite 180; Sonosite, Bothell, WA, USA), using a linear 3- to 11-MHz transducer with ranges between 3 and 5 cm. All sonographic examinations were performed by a single experienced shoulder surgeon, who performed an average of 15 shoulder US examinations per week for more than 10 years. The examination was performed with the patient in the supine position on the examination table. A continuous complete rotator cuff was considered to be intact in the absence of a focal defect or sign of retraction or avulsion, while a partial-thickness tear was registered when a focal defect was present either on the articular surface or on the bursal surface of the rotator cuff [32]. The contralateral shoulder was also assessed to compare the rotator cuff echogenicity and the presence of calcifications.

As a primary endpoint, the pain was quantified using the VAS scale with scores ranging from 0 (no pain) to 10 (worst imaginable pain). As a secondary endpoint, the functional recovery was monitored using the Constant Score with scores ranging from 0 (most disability) to 100 points (least disability) and the Disability of the Arm, Shoulder and Hand Score (DASH) with scores ranging from 0 (no disability) to 100 (most severe disability).

### Statistical analysis

The collected data were analyzed using SPSS (v 23; IBM Inc). Descriptive statistics were calculated for the overall sample and follow-up and pathology. Categorical variables were presented as numbers or percentages. Continuous variables were presented as median and range when non-normally distributed or as mean and standard deviation for normally distributed variables. The Shapiro–Wilk test was used to test for the normality of the data. Mann–Whitney U tests or Kruskal–Wallis tests for group comparisons were conducted for follow-ups and pathologies since the variables were not normally distributed. A *p*-value of 0.05 was considered statistically significant.

## Results

The main data of the study are summarized in Table 1. Seventy-two patients (35 male, mean age = 43.75 ± 10.82, range 21–58 years old) were enrolled in the study group, whereas seventy patients (32 male; mean age = 41.29 ± 13.85, range 20–65 years old) were enrolled in the control group.

**Table 1:** Main data of the study

	Study group (*n* = 72)	Control group (*n* = 70)
*Age (year)*		
Mean ± SD	43.75 ± 10.82	41.29 ± 13.85
Range	21–58	20–65
*Gender*		
Male *n* (%)	35 (46.6%)	32 (45.7%)
Female *n* (%)	40 (53.4%)	38 (54.3%)
*BMI (Kg/m2)*		
Mean ± SD	25.4 ± 3.9	24.2 ± 3.3
Range	21.5—29.3	20.9 – 27.5
*Side*		
Left *n* (%)	42 (56%)	37 (52.8%)
Right *n* (%)	33 (44%)	86 (47.2%)

**Fig. 2:**
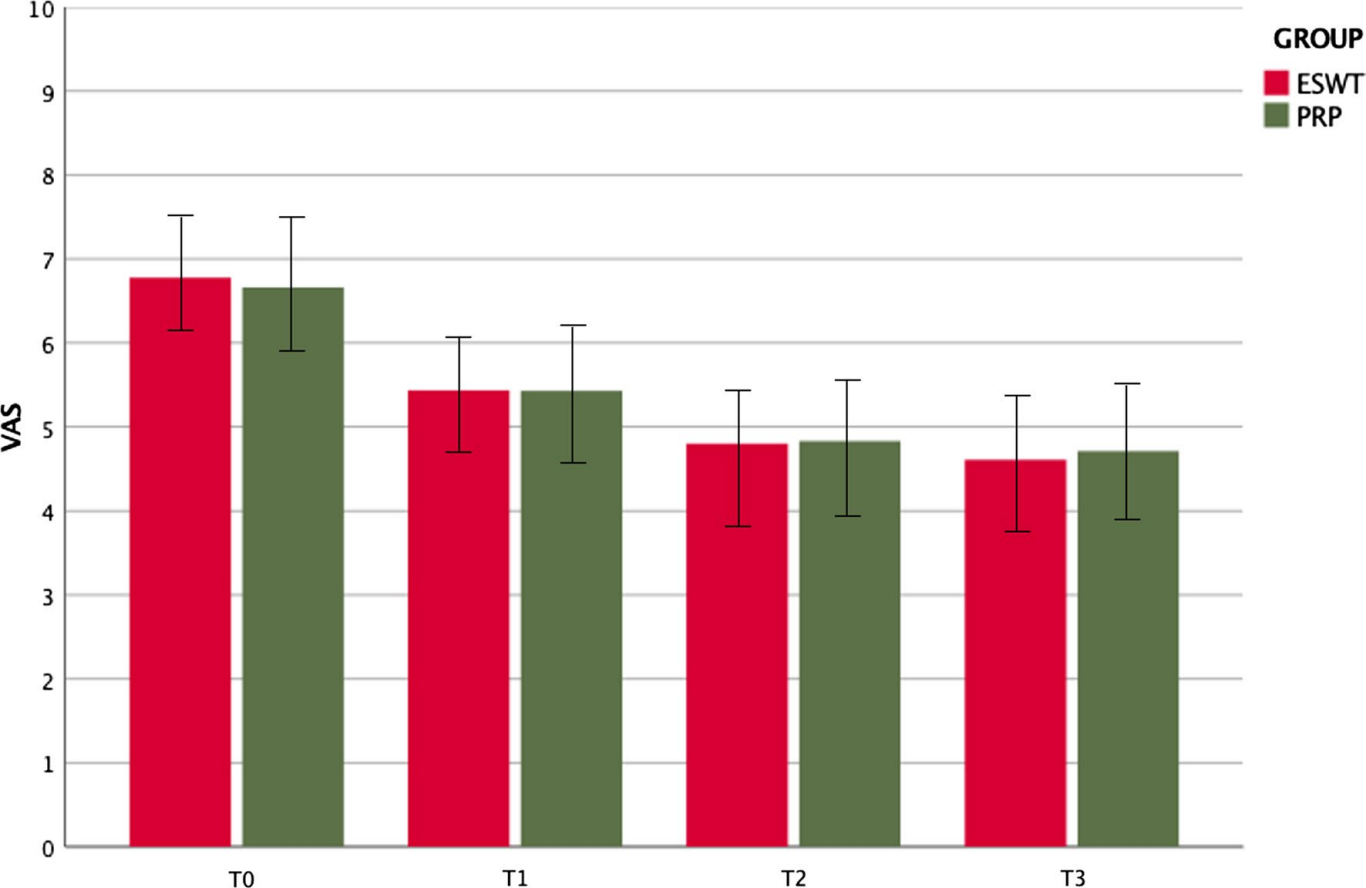
Comparison between evaluation times of VAS score

In the study group, the mean VAS score was 6.71 ± 1.71 at T0, 5.16 ± 1.66 at T1, 4.83 ± 1.79 at T2, and 4.7 ± 1.85 at T3. A significant improvement was observed between T0 and T1 (*p* = 0.001) and between T0 and T3 (*p* = 0.001) (Fig. 2). In the control group, the mean VAS score was 6.77 ± 1.46 at T0, 5.43 ± 1.47 at T1, 4.79 ± 1.74 at T2, 4.61 ± 1.70 at T3. The comparative analysis between groups showed no statistical differences at follow-ups (*p* = 0.66 at T0; *p* = 0.96 at T1; *p* = 0.96 at T2 and *p* = 0.71 at T3) (Fig. 2).

**Fig. 3:**
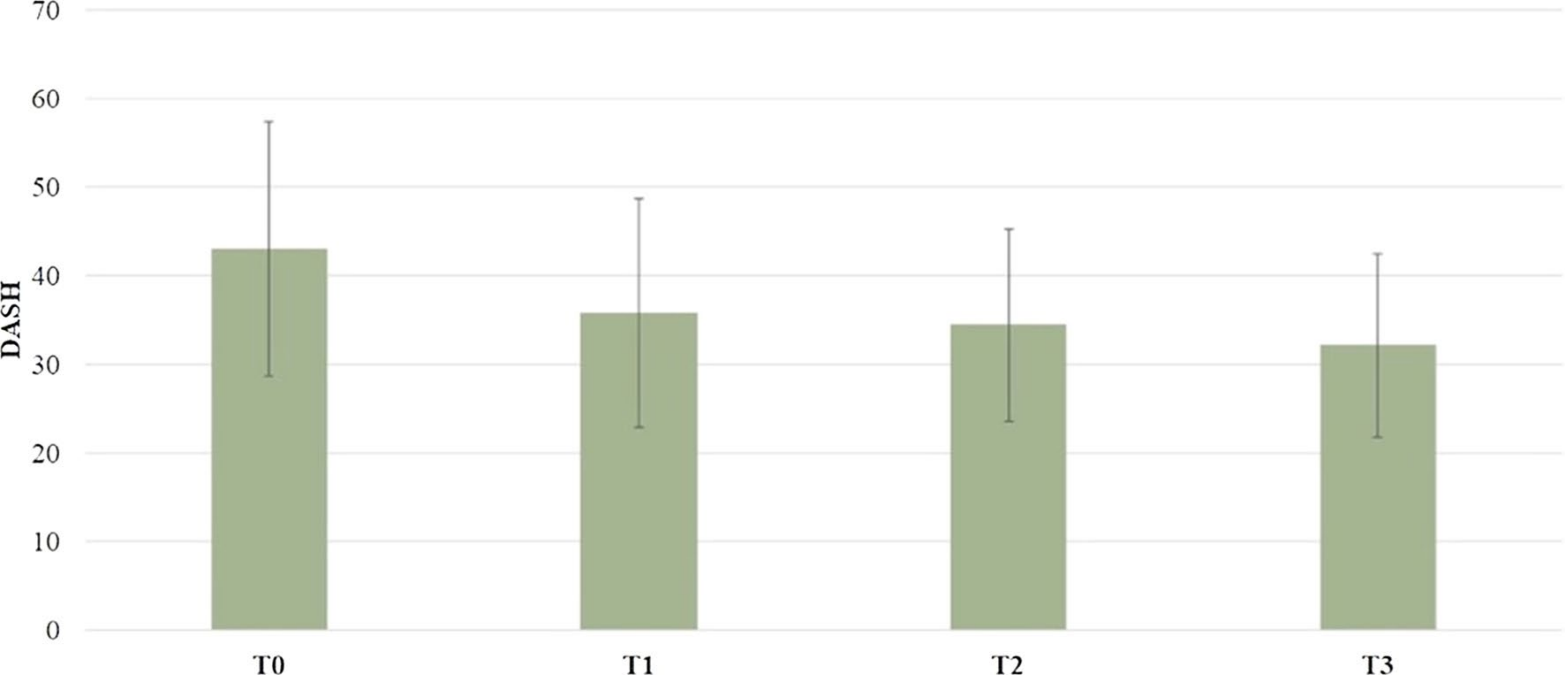
Comparison between evaluation times of DASH score

In the study group, the mean DASH score was 43.04 ± 14.29 at T0, 35.89 ± 12.87 at T1, 34.45 ± 10.88 at T2, 32.18 ± 10.29 at T3. A significant statistical difference emerged between T0 and T1 (*p* = 0.017), and T0 and T3 (*p* = 0.001) (Fig. 3).

**Fig. 4:**
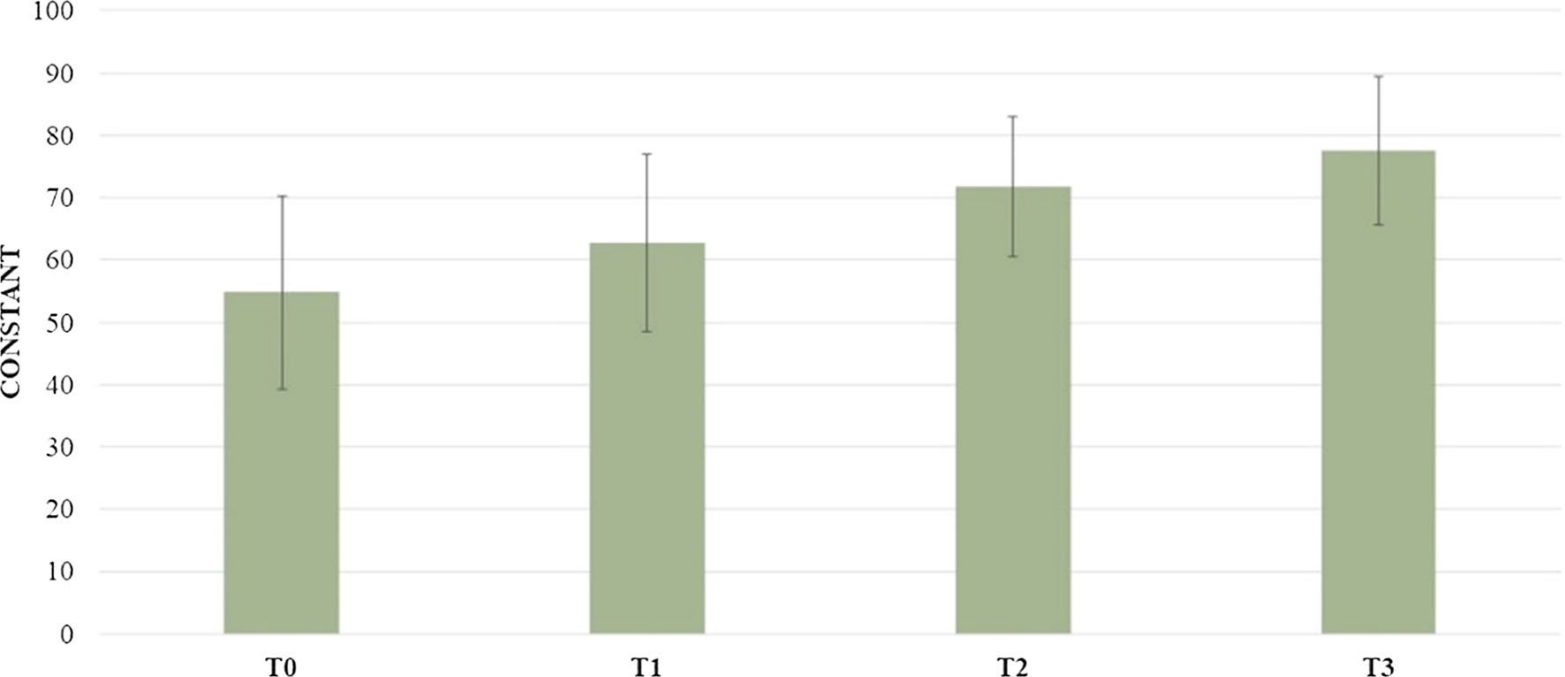
Comparison between evaluation times of constant shoulder score

The mean Constant Shoulder score was 54.79 ± 15.46 at T0, 62.79 ± 14.27 at T1, 71.79 ± 11.24 at T2, 77.54 ± 11.84 at T3. Statistical differences emerged between T0 and T1 (*p* = 0.009), T1 and T2 (*p* = 0.001), and T0 and T3 (*p* = 0.001) (Fig. 4).

The items of the Constant score are analyzed in detail and summarized in Table 2. Statistical significance difference emerged between T0 and T3 in each subgroup, except in subgroup “work” with a *p*-value of 0.103.

**Table 2:** Constant score items in the study group at follow-ups

	T0 (M ± SD)	T1 (M ± SD)	T2 (M ± SD)	T3 (M ± SD)
Pain	3.33 ± 4.03	7.29 ± 4.6	8.75 ± 3.92	10.2 ± 4.24
Sleep	0.58 ± 0.91	0.91 ± 1	1.33 ± 0.95	1.41 ± 0.91
Recreational/Sport	1.66 ± 1.99	1.83 ± 2.01	3 ± 1.75	3.16 ± 1.64
Work	1.83 ± 2.01	1 ± 1.75	1.83 ± 2.01	2.5 ± 1.95
Arm positioning	6 ± 2.73	6.5 ± 2.55	7.25 ± 2.24	8.16 ± 2.25
Strength of Abduction	14.54 ± 6.46	15.58 ± 6.24	17.79 ± 5.44	18.83 ± 5.23
Forward flexion	7.16 ± 2.09	7.91 ± 1.79	8.5 ± 1.67	8.83 ± 1.41
Lateral elevation	6.5 ± 2.49	7.41 ± 2.22	7.91 ± 2.14	7.91 ± 1.88
External rotation	7 ± 2.54	7.75 ± 2.75	8.25 ± 2.62	8.58 ± 2.22
Internal rotation	6.16 ± 2.73	6.58 ± 2.76	7.16 ± 2.39	7.91 ± 1.97
Total	54.79 ± 15.46	62.79 ± 14.27	71.79 ± 11.24	77.54 ± 11.84

In the study group, the ultrasound (US) examination at T3 showed a reduction in hypoechoic areas, an increase in tendon hyperechogenicity and a reduction in vascularity compared to T0.

## Discussion and conclusions

PRP injections have been used in chronic tendon injuries for more than a decade [1–3, 14, 15]. As an autologous biologic treatment for musculoskeletal injuries, PRP has several advantages: it is simple to plan, has few side effects, and presents several therapeutic effects. Although various protocols and application methods have been described for PRP, there is still no consensus on the optimal PRP preparation, dose, volume and posology [16].

Chen et al. [11] in a recent meta-analysis including 37 studies, reported 17 studies out of 37 (45.95%) did not activate PRP. All but 3 of the 18 studies that included an activating agent used some form of calcium (CaCl_2_, calcium gluconate, or CaCl_2_ and thrombin) to activate PRP [11]. Two studies used thrombin alone and one study used Type I collagen [11].

Few studies attempted to quantify platelets (40.5%) or leukocytes (24.3%), but the majority reported the volume of PRP injection, the mean volume was 5.30 ± 5.76 mL. The different amounts of volume may depend on the site of infiltration (greater for intra-articular infiltration of the knee, smaller for tendon/peritendinous tissue). In our clinical practice, we use a concentration of 7% (with no activating agent) and ml 4 for one-shot infiltrations for supraspinatus tendinopathy.

Some authors use PRP as augmentation after shoulder arthroscopy with positive effects on pain and healing [17, 18]. Other authors have shown unsatisfactory results of this procedure at 1-year follow-up, compared to the control group [19].

In the literature, there are no homogeneous results both at short-term and long-term follow-ups for PRP injections for supraspinatus tendinopathy or other rotator cuff pathologies. Kesikburun et al. [20] and Hurley et al. [21] highlight poor results, others believe that the use of PRP is particularly effective since it promotes biological cell regeneration, reduces inflammation and avoids a surgical procedure [22–25].

In the present study, we enrolled 72 patients (male: 35) no statistically significant gender differences emerged in the clinical scores assessment at the various follow-ups. All the clinical scores showed a significant improvement as early as 1 month after infiltration; the mean VAS score underwent the greatest improvement. There was a gradual and slow improvement even at 3 months and 6 months of follow-up. The improvement in scores was almost always statistically significant compared to T0 but rarely between T1 and T2 and between T2 and T3. PRP intratendinous injections showed a non-inferiority compared to ESWT in the management of supraspinatus tendinopathy.

According to the findings of the present study, the most important element for PRP infiltrative therapy success is the use of adequate inclusion and exclusion criteria. Patients with Supraspinatus tendonitis, intratendinous calcifications and partial tendon tears should be included, while patients with complete supraspinatus lesions or other several rotator cuff tendons tears should be completely excluded. Furthermore, patients with a particular anatomical structure of the acromion clavicle joint, facilitating continuous damage of the supraspinatus tendon should not be candidates for PRP injections, but arthroscopic acromioplasty should be performed in these cases.

Subacromial PRP infiltrative therapy can be also associated with Sodium Hyaluronate to obtain better results as suggested by Cai et al. [26].

Albert JD et al. [34] reported symptoms of improvement in patients with calcifying tendinitis of the rotator cuff as early as a 3-month follow-up with ESWT. Van Kampen DA et al. [35] confirmed previous results in a study recruiting one hundred and ten patients. Therefore, nowadays the ESWT has been recognized as a valid and safe treatment for supraspinatus tendinosis and other cuff tear pathology [30]. We reported non-inferiority of single shoulder injection of PRP if compared with ESWT at each follow-up. These results are in-line with the current literature [36].

This study has some limitations. First, the sample size is quite small; second, there is no comparison between single and multiple injections, third, there is not an MRI comparison before and after the injection and the improvement evidenced by the US examination cannot be objectively demonstrated. Additionally, the follow-up period was short- and long-term effectiveness was therefore not assessed. On the other hand, a strong point of this work is the selection of treated patients, respecting stringent inclusion and exclusion criteria.

In conclusion, considering the benefits of PRP, the one-shot infiltration in the tendon site of the supraspinatus muscle and peritendinous at the subacromial space represents a valid conservative treatment to reduce pain, improve Quality of life and functional scores even at midterm of 6 months follow-up. Therefore, further investigation and studies are needed in the future about this topic.
